# LyeTxI-b, a Synthetic Peptide Derived From a Spider Venom, Is Highly Active in Triple-Negative Breast Cancer Cells and Acts Synergistically With Cisplatin

**DOI:** 10.3389/fmolb.2022.876833

**Published:** 2022-05-04

**Authors:** Joaquim Teixeira de Avelar Júnior, Edleusa Lima-Batista, Célio José Castro Junior, Adriano Monteiro de Castro Pimenta, Raquel Gouvêa Dos Santos, Elaine Maria Souza-Fagundes, Maria Elena De Lima

**Affiliations:** ^1^ Departamento de Bioquímica e Imunologia, Instituto de Ciências Biológicas Universidade Federal de Minas Gerais, Belo Horizonte, Brazil; ^2^ Programa de Pós-Graduação em Medicina e Biomedicina da Santa Casa de Belo Horizonte, Belo Horizonte, Brazil; ^3^ Centro de Desenvolvimento da Tecnologia Nuclear (CDTN), Belo Horizonte, Brazil; ^4^ Departamento de Fisiologia e Biofísica, Instituto de Ciências Biológicas Universidade Federal de Minas Gerais, Belo Horizonte, Brazil

**Keywords:** antitumoral peptide, breast cancer, MDA-MB-231, *Lycosa erythrognatha*, drug combination, isobolographic analysis, LyeTxI-b peptide

## Abstract

Breast cancer is the most common cancer that affects women globally and is among the leading cause of women’s death. Triple-negative breast cancer is more difficult to treat because hormone therapy is not available for this subset of cancer. The well-established therapy against triple-negative breast cancer is mainly based on surgery, chemotherapy, and immunotherapy. Among the drugs used in the therapy are cisplatin and carboplatin. However, they cause severe toxicity to the kidneys and brain and cause nausea. Therefore, it is urgent to propose new chemotherapy techniques that provide new treatment options to patients affected by this disease. Nowadays, peptide drugs are emerging as a class of promising new anticancer agents due to their lytic nature and, apparently, a minor drug resistance compared to other conventional drugs (reviewed in Jafari et al., 2022). We have recently reported the cytotoxic effect of the antimicrobial peptide LyeTx I-b against glioblastoma cells (Abdel-Salam et al., 2019). In this research, we demonstrated the cytotoxic effect of the peptide LyeTx I-b, alone and combined with cisplatin, against triple-negative cell lines (MDA-MD-231). LyeTx-I-b showed a selectivity index 70-fold higher than cisplatin. The peptide:cisplatin combination (P:C) 1:1 presented a synergistic effect on the cell death and a selective index value 16 times greater than the cisplatin alone treatment. Therefore, an equi-effective reduction of cisplatin can be reached in the presence of LyeTx I-b. Cells treated with P:C combinations were arrested in the G2/M cell cycle phase and showed positive staining for acridine orange, which was inhibited by bafilomycin A1, indicating autophagic cell death (ACD) as a probable cell death mechanism. Furthermore, Western blot experiments indicated a decrease in P21 expression and AKT phosphorylation. The decrease in AKT phosphorylation is indicative of ACD. However, other studies are still necessary to better elucidate the pathways involved in the cell death mechanism induced by the peptide and the drug combinations. These findings confirmed that the peptide LyeTx I-b seems to be a good candidate for combined chemotherapy to treat breast cancer. In addition, *in vivo* studies are essential to validate the use of LyeTx I-b as a therapeutic drug candidate, alone and/or combined with cisplatin.

## 1 Introduction

Breast cancer is the leading cause of cancer in women and contributes to almost 25% of all cases and 15% of all cancer deaths (Bray et al., 2018). The subtype triple-negative, responsible for 15–20% of all breast cancer cases (Anders et al., 2016), is more aggressive and difficult to treat (Gonçalves Jr et al., 2018). Due to the lack of estrogen, progesterone, and HER2 receptors (Bauer et al., 2007), and the inactivation of the BRCA1 gene (Anders et al., 2016), hormonal therapy, one of the most common treatments for breast cancer (Puhalla et al., 2012; Wahba and El-Hadaad, 2015), is not available, worsening the prognosis of the disease compared to other types of breast cancers (Ismail-Khan and Bui, 2010). Indeed, the treatment is only based on chemotherapy and surgery (Wahba and El-Hadaad, 2015), even though radiotherapy can be employed in some cases (Wahba and El-Hadaad, 2015). In addition, among the different types of breast cancers, triple-negative is the most immunogenic tumor. Among the factors that confer this immunogenicity are the expression of programmed cell death protein 1 (PD-1) and programmed cell death ligand 1 (PD-L1). PD-1 is critical in inhibiting immune responses. PD-L1 is considered an inhibitory cofactor of the immune response that, by binding to PD-1 present in PD1-positive cells, suppresses the immune response and induces death by apoptosis (Han et al., 2020). Moreover, PD-L1 favors the escape of tumor cells from the immune response. Therefore, inhibitors of the checkpoint inhibitor class PD1/D-L1 have been shown to be therapeutic options for treating triple-negative breast cancer. Inhibitors such as pembrolizumab, an anti-PD-1 inhibitor, and atezolizumab (PD-L1) have been approved by the FDA for cancer treatment (Han et al., 2020; Heeke and Tan, 2021). Since targeting signaling molecules is virtually difficult for triple-negative breast cancer treatment, discovering new therapeutic drugs or combining two or more drugs and searching for synergistic benefits may be especially valuable in triple-negative breast cancer treatment (Jhan and Andrechek, 2017). In this scenario, it is fundamental to assess the potential of combination treatment (Jhan and Andrechek, 2017).

Platinum compounds (e.g., cisplatin and carboplatin) are chemotherapy agents used to deal with triple-negative breast cancers (Wahba and El-Hadaad, 2015). They cause DNA damage (Dasari and Tchounwou, 2014) by chemically binding to the DNA and blocking replication and transcription events (Fuertes et al., 2003; Wang and Lippard, 2005; Dasari and Tchounwou, 2014; Melnikov et al., 2016). Nonetheless, severe side effects, such as vomiting (Kottschade et al., 2016), nephrotoxicity (Manohar and Leung, 2018), and drug resistance, limit their efficacy (Jhan and Andrechek, 2017; Rocha et al., 2018; De Luca et al., 2019; Gomes et al., 2019). A strategy to circumvent these issues would be the combined therapy, in which different anticancer agents with different targets and objectives are employed. In this approach, different chemotherapeutic agents are administered, targeting different populations. Therefore, the probability of killing all tumor cells (Palmer and Sorger, 2017), reducing resistance (Chou, 2006; Foucquier and Guedj, 2015), and decreasing toxicity compared to monotherapy increases (Mokhtari et al., 2017). The combination of regular chemotherapy compounds and anticancer peptides is well described in the literature (Hazama et al., 2014; Su et al., 2014; Qiao et al., 2018).

LyeTx I-Des-His (or LyeTx I-b) is a synthetic antimicrobial peptide derived from LyeTx I, a peptide purified from the venom of the spider *Lycosa erythrognatha* (Santos et al., 2010; Reis et al., 2018). Recently, we demonstrated that LyeTx I-b has cytotoxic activity against the glioblastoma cell model (Abdel-Salam et al., 2019). As demonstrated using microorganisms, it is believed that this peptide acts by a membranolytic mechanism (Reis et al., 2018; Abdel-Salam et al., 2019) since the cancer cell membrane is also negatively charged (Chen et al., 2016). However, other mechanisms of action of this peptide in cancer cells cannot be excluded, and studies have been conducted to elucidate these mechanisms. A combination of a platinum compound and an antimicrobial peptide showing cytotoxicity for tumoral cells is a rational choice that explores different mechanisms of action against these cells.

Therefore, the main goal of this work was to evaluate the activity of LyeTx I-b and its combination with cisplatin against the breast cancer cell line MDA-MB-231.

## 2 Materials and Methods

### 2.1 Materials

The cell lines used in this study were MDA-MB-231 (ATCC#: HTB-26) and HEK-293 (ATCC#: CRL-1573). The culture medium used was Dulbecco’s modified Eagle’s medium (DMEM) supplemented with fetal bovine serum (10%) (Invitrogen Company, Carlsbad, California, United States). The peptide LyeTx I-b (sequence: CH_3_CO-IWLTALKFLGKNLGKLAKQQLAKL-NH_2_) was purchased from GenOne Company (Rio de Janeiro, Brazil). Cisplatin, propidium iodide, Acridine orange, and Hoechst dye were purchased from Sigma-Aldrich (St. Louis, Missouri, United States). All reagents used in this research were of analytical grade.

### 2.2 Methods

#### 2.2.1 Cell Culture

The cell lines MDA-MB-231 and HEK-293 were cultured in DMEM supplemented with fetal bovine serum (10%) and incubated at 37°C, in a 5% CO_2_ atmosphere.

#### 2.2.2 Cell Viability Assay

Cytotoxicity was evaluated using the tetrazolium dye (MTT) assay (Mosmann, 1983; Abdel-Salam et al., 2019). MTT at 5 mg/ml was added to the treated cells and incubated for 4 h at 37°C and 5% CO_2_. After this time, the supernatant was carefully removed, and the formazan crystals were solubilized with 100 µl of 2-propanol containing 0.04 m HCl and read at 595 nm in a Varioskan Lux apparatus. The IC_50_ values were calculated with GraphPad Prism v. 5.01 software using nonlinear regression. Results were expressed as a percentage of cell survival or death.

#### 2.2.3 Cell Treatment

MDA-MB-231 or HEK-293 cells were plated (10,000 cells/well) in 96-well plates and incubated for 24 h at 37°C and 5% CO_2_. Cells were divided into five groups of treatments: monotherapies with increasing concentrations (0.39–12.5 µM) of LyeTx I-b or cisplatin (1.17–300 µM) and a combination of three different ratios of LyeTx I-b peptide (P) and cisplatin (C). Molar ratios (P:C) were based on a maximum concentration of 12.5 µM of LyeTx I-b in all treatments and twofold serial dilutions to 1:1, 3:1, and 1:3 combinations, as specified in Table 1. The combined effect of both drugs was analyzed using isobolograms, as previously reported (Tallarida, 2000, 2016). Graphic isobolograms were created using the IC_50_ values and a statistical test was performed (*t*-test; statistical significance of 95%) (Tallarida, 2000, 2016; Chou, 2006) using Microsoft Excel.

**TABLE 1:** Concentrations used in each combination to perform isobologram analyses.

Proportion 1:1 P:C		Proportion 3:1 P:C		Proportion 1:3 P:C	
LyeTx I-b (µM)	Cisplatin (µM)	LyeTx I-b (µM)	Cisplatin (µM)	LyeTx I-b (µM)	Cisplatin (µM)
12.5	12.5	12.5	4.16	12.5	37.5
6.25	6.25	6.25	2.08	6.25	18.75
3.12	3.12	3.12	1.04	3.12	9.37
1.56	1.56	1.56	0.52	1.56	4.68
0.78	0.78	0.78	0.26	0.78	2.34
0.39	0.39	0.39	0.13	0.39	1.17

#### 2.2.4 Cell Cycle

The DNA content of cells was evaluated by the Nicoletti method, based on fluorescence that separates different populations in distinct cell cycle phases and evaluates DNA fragmentation (Nicoletti et al., 1991). MDA-MD-231 cells were plated (300,000/per well in 6-well plates) and incubated for 48 h at an IC_50_ concentration found for each combination and each compound studied. After each treatment, the cells were trypsinized and washed; the recovered medium was centrifuged at 2,000 g for 5 min. The supernatant was discarded, and the recovered precipitate was treated with 300 µl of an HFS- solution, containing 0.1% (w/v) sodium citrate; 0.1% (v/v) Triton X-100, and 50 µg/ml propidium iodide for 1 h. Then, the fluorescence emitted by the dye used was read on a FACScan using the 585/42 filter.

#### 2.2.5 Acridine Orange Incorporation Assay

The MDA-MB-231 cells were plated (300,000/well in 96-well plates), incubated for 48 h at an IC_50_ concentration found for each combination and each compound studied, in the absence or the presence of 10 nm bafilomycin A1 pre-treated for 1 h. After each treatment, the cells were trypsinized and washed with the recovered medium centrifuged at 2,000 g for 5 min. After that, the solution was discarded, and 300 µl of a solution containing 5 µg/ml acridine orange in PBS was added to the cells (Murugan and Amaravadi, 2016). The preparation obtained was read in the FACScan using the 530/30 and 670LP filters.

The data obtained were analyzed with FlowJo 10.0 software.

#### 2.2.6 Immunoblotting

Following the drug treatments, MDA-MB-231 cells were lysed in RIPA buffer (0.15 m NaCl, 0.05 m Tris-HCl, pH 7.2, 0.05 m EDTA, 1% Nonidet P40, 1% Triton X-100, 0.5% sodium deoxycholate, 0.1% SDS) containing protease inhibitors (1.0 mm AEBSF and 10.0 µg/ml of both leupeptin and aprotinin). The total cellular protein (50 µg) for each sample was subjected to SDS-PAGE (10%), followed by electroblotting onto nitrocellulose membranes. Membranes were blocked with 5% BSA in wash buffer (150.0 mm NaCl, 10.0 mm Tris-HCl, pH 7.4, and 0.1% Tween 20) for 2 h. Then, they were incubated with either rabbit anti-phospho AKT1 (S473, DB Biotech # DB 127 0.1; 1:1,000), rabbit anti-phospho ERK1/2 (Thr202/Tyr204, Invitrogen S.812.9 # MA5-15173; 1:1,000), rabbit anti-p21 (F-5, Santa Cruz # SC 6246; 1:300), mouse anti-p53 (DO-1, Santa Cruz # SC 126; 1:1,000), or antibodies in wash buffer containing 5% BSA overnight at 4°C. Membranes were rinsed three times with wash buffer and then incubated with the secondary peroxidase-conjugated anti-rabbit IgG antibody (Millipore, # AP307P) diluted 1:3,000 or anti-mouse IgG (Invitrogen, # 626520; 1:2000) in wash buffer containing 5% BSA for 1 h. Then, they were rinsed three times with wash buffer, incubated with Western blot detection reagents (Millipore, Luminata Forte Western HRP Substrate, # WBLUF0500), and scanned and analyzed using ImageQuant LAS 4000 (GE Healthcare). Membranes were stripped and incubated with either rabbit anti-AKT (DB Biotech, # DB 126 0.1; 1:1,000), rabbit anti-ERK1/2 (Invitrogen K.913.4, # MA5-1,534; 1:1,000) or rabbit anti-*β* actin (Sigma RM112, # MATB523; 1:3,000) in wash buffer containing 5% BSA overnight for 4°C. Membranes were rinsed three times with the wash buffer and then incubated with the secondary peroxidase-conjugated anti-rabbit IgG antibody (Millipore, # AP307P) diluted 1:3,000 or anti-mouse IgG (Invitrogen, # 626520), (1:2000) in wash buffer containing 5% BSA for 1 h and probed with the secondary antibody anti-rabbit IgG diluted 1:5,000 to determine total AKT, ERK1/2, and p21 and p53 expression. Non-saturated, immunoreactive AKT, ERK1/2, p21, and p53 bands were quantified by scanning densitometry. The immuno-band intensity was calculated using ImageJ software. The number of AKT and ERK1/2 phospho-bands was divided by the number of pixels of total AKT and ERK1/2 to normalize the phosphorylation levels of kinases to total kinase expression and p21 and p53. Bands were divided by the number of pixels of *β* actin to normalize the expression levels.

#### 2.2.7 Statistical Analysis

Data were analyzed by the one-way ANOVA (Analysis of Variance) statistical test, followed by the Bonferroni *post*-*hoc* test. A statistical significance of 95% for all tests was considered.

## 3 Results

### 3.1 Cell Viability Evaluation

**FIGURE 1:**
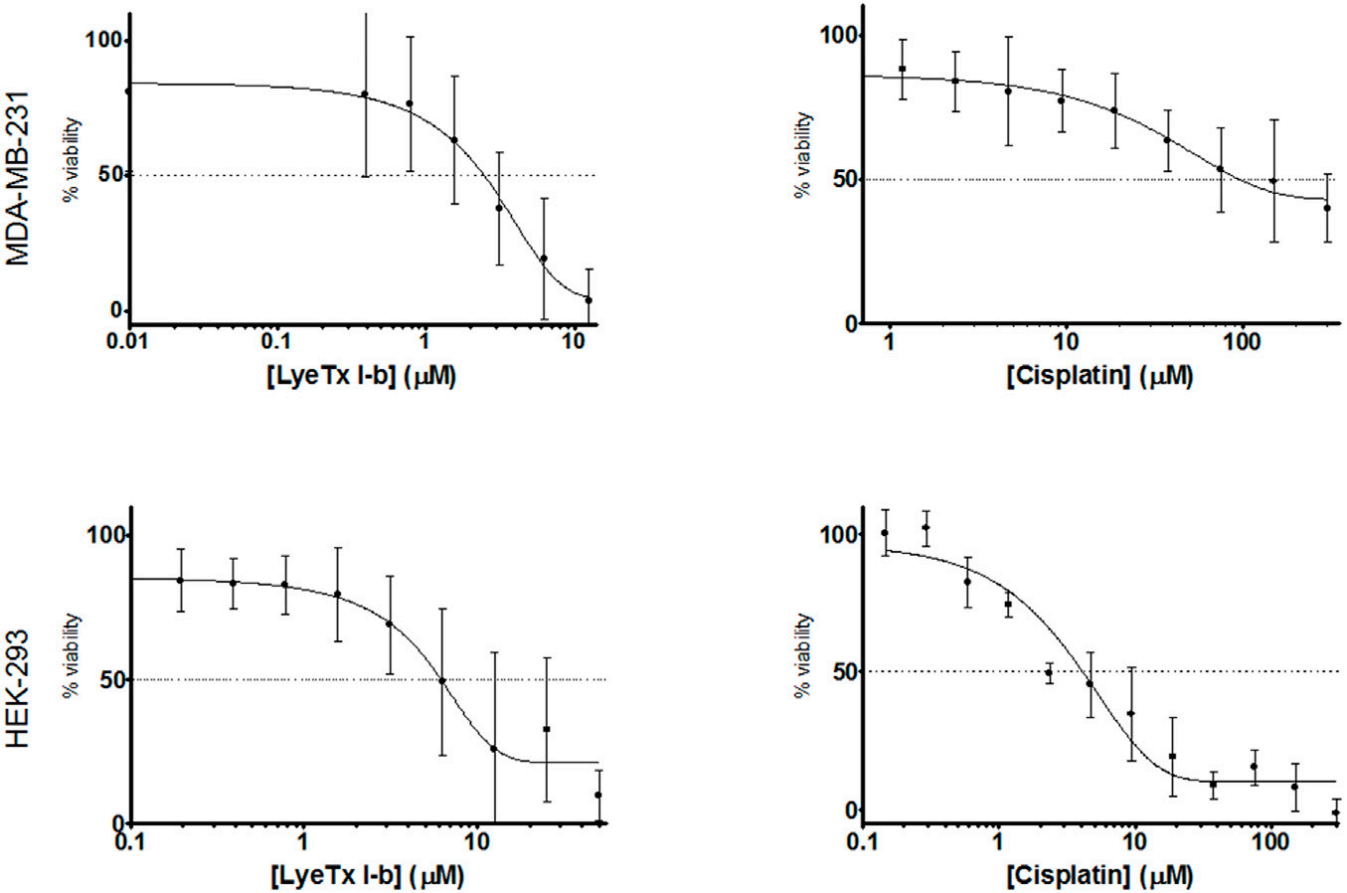
Comparison of cytotoxicity of LyeTx I-b or cisplatin, as indicated on the *x*-axis, in MDA-MB-231 (a, b, *n* = 8) and in HEK-293 cells (c, d, *n* = 3)). Experiments were performed after 48 h of treatment and evaluated by MTT assay.

LyeTx I-b and cisplatin treatments induced cytotoxicity in a concentration-dependent manner (Figure 1) against MDA-MB-231 (triple-negative breast cancer cells) and HEK-293 (fetal kidney cells). The IC_50_ values of LyeTx I-b were 2.47 µM against MDA-MB-231 and 7.88 µM against HEK-293. The IC_50_ values of cisplatin were 94.69 and 3.96 µM against MDA-MB-231 and HEK-293, respectively.

The selectivity index (SI) value ( the HEK [IC_50_]/MDA [IC_50_] ratio ) for the peptide LyeTx I-b was 3.19, which is 77 times higher than that of cisplatin (0.041).

### 3.2 Isobolographic Analysis of LyeTx I-b Combined With Cisplatin Demonstrated Synergism for One of the Studied Doses

Isobolographic analysis was performed to evaluate the effects of combined drug treatments against MDA-MB-231 and HEK-293.

**FIGURE 2:**
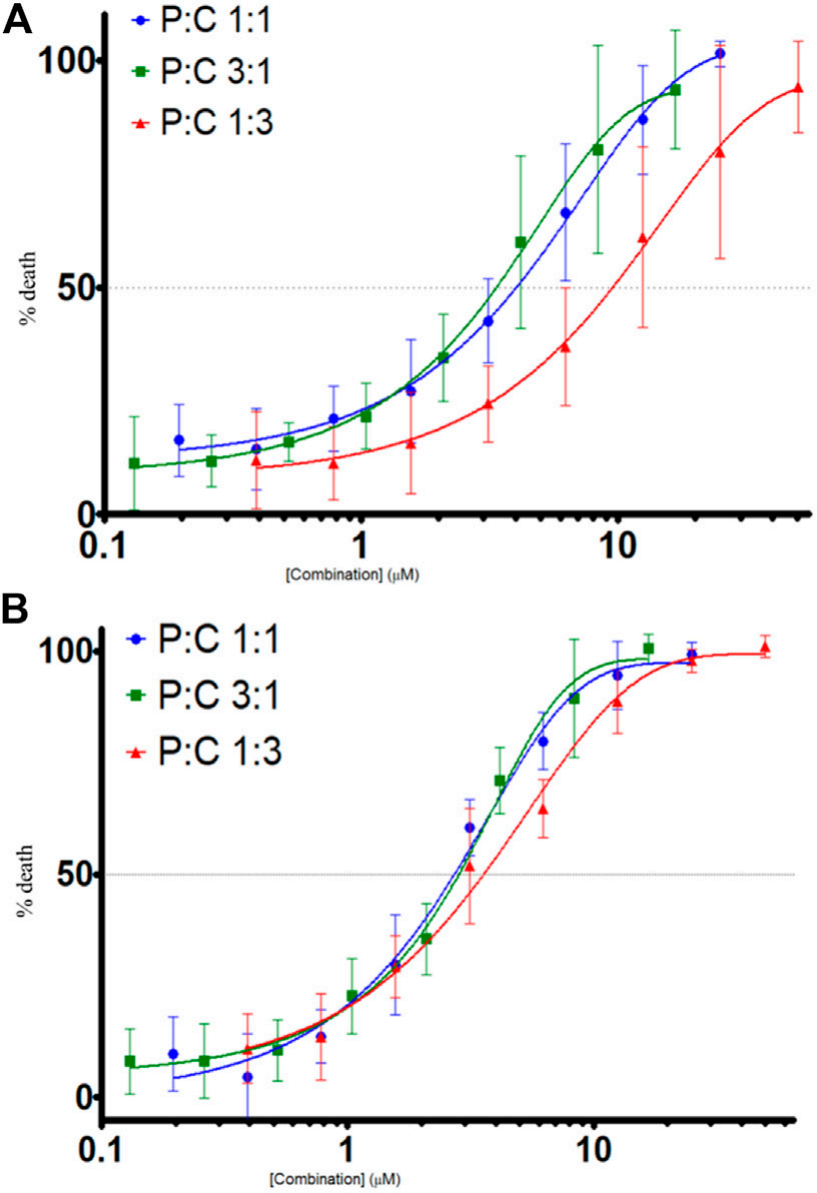
Cell death evaluation (%) by combined treatments of LyeTx I-b and cisplatin, in different proportions as indicated, against MDA-MB-231, *n* = 6 **(A)** and HEK-293 cells, *n* = 5 **(B)**. Cell death was evaluated by MTT assay, after 48 h of treatments with the compounds.

The proportions of LyeTx I-b and cisplatin (P:C = 1:1, 3:1, and 1:3) were evaluated against MDA-MB-231 and HEK-293 cells, resulting in concentration-dependent curves (Figure 2) for each proportion. These curves were made using the following equation: % death = 100%–% viability. The IC_50_ values found for each treatment against the cells and the SI values are shown in Table 2.

**TABLE 2:** IC_50_ values of the compounds in different proportions.

Proportion used	MDA-MB-231 IC_50_	HEK-293 IC_50_	SI
LyeTxI-b cisplatin 1:1	3.97	2.67	0.67
LyeTxI-b cisplatin 3:1	3.37 µM	2.87	0.85
LyeTxI-b cisplatin 1:3	9.57 µM	3.57	0.37

SI (selective index) represents the ratio between the IC_50_ values of compounds and the cells (HEK-293/MDA-MB 231).

**FIGURE 3:**
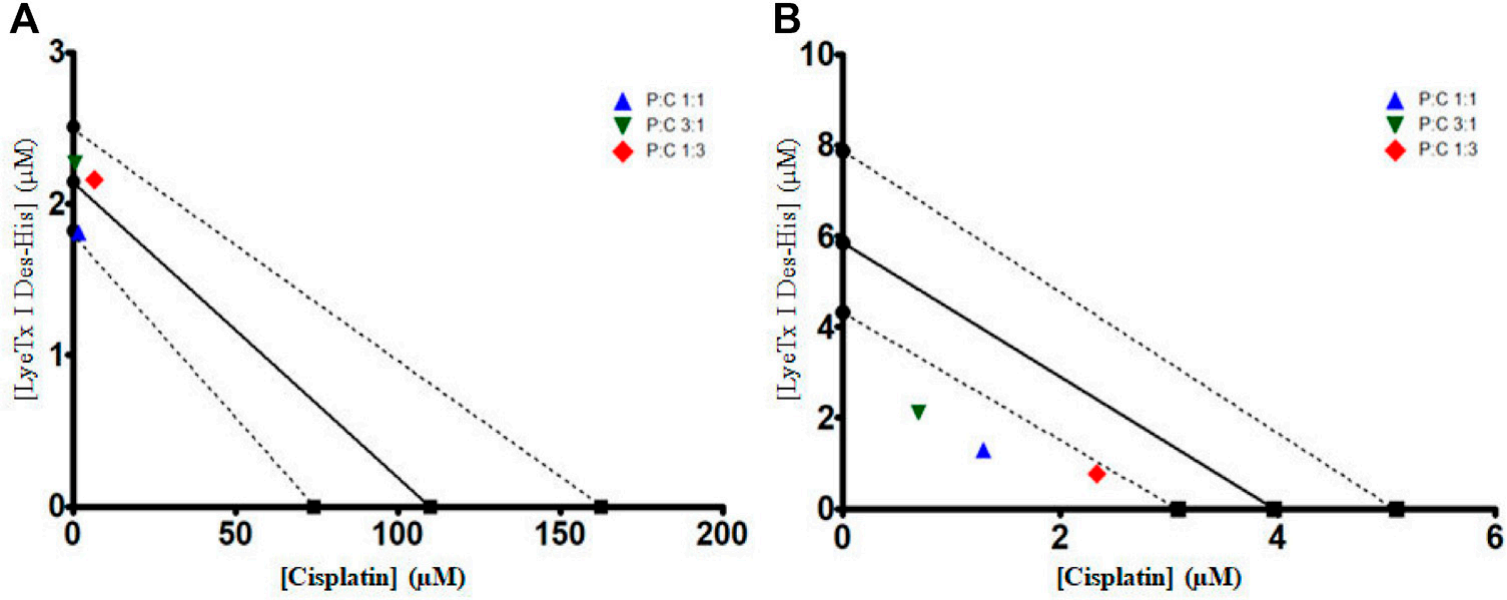
IC_50_ isobolograms to each combined treatment of LyeTx I-b and cisplatin, in different proportions (as indicated), against MDA-MB-231 **(A)** and HEK-293 cells **(B)**. A *t*-test was performed between each experimental IC_50_ and the theoretical IC_50_, showing that against MDA-MB-231 cells, combined treatment 1:1 (P:C) was statistically different from the theoretical value. For HEK-293 cells, all combined treatments were statistically different from their respective theoretical values.

The isobolographic statistical analysis considering the IC_50_ of each treatment revealed that the combination of LyeTx I-b with cisplatin (proportion 1:1) against MDA-MB-231 provoked a synergistic effect. In contrast, the other two combinations (3:1 and 1:3) presented an additive effect. On the other hand, when tested against HEK-293 cells, the compounds in all the proportions studied (i.e., 1:1, 3:1, and 1:3) showed a synergistic effect, which was demonstrated by the *t*-test. Figure 3 shows the isobolograms of both cell lines. Regarding the SI values (the ratio between HEK-293 IC_50_ and MDA-MB-231 IC_50_; HEK [IC_50_]/MDA [IC_50_]), it was found that the combinations 1:1, 3:1, and 1:3 presented SI of 0.67, 0.85, and 0.37, respectively (Table 2), which were higher than that of cisplatin (0.041).

### 3.3 Cell Cycle Progression Indicated Arrest in the Cell Cycle

**FIGURE 4:**
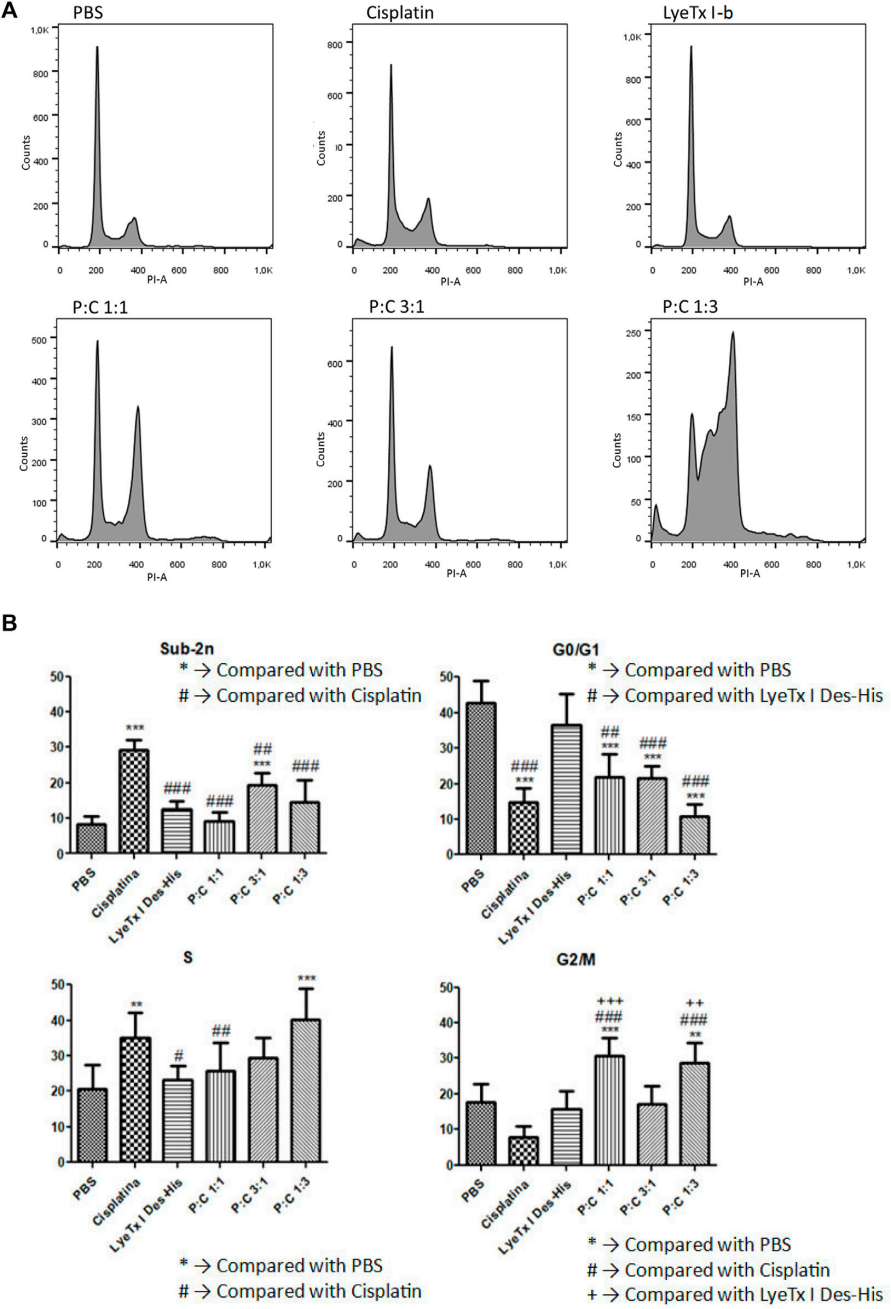
Cell cycle evaluation by the DNA content marked with propidium iodide after 48 h of treatment against MDA-MB-231 cells. **(A)** A representative histogram showing the results to each treatment for their respective IC_50_ values . **(B)** Phase quantification (*n* = 4) to each treatment for their respective IC_50_ values. Statistical tests were performed by one-way ANOVA displayed by the codes in figure. #*p* < 0.05; **, ++, ## *p* < 0.01 and ***, +++, ### *p* < 0.001.

The cell cycle analysis indicated different progressions in the distinct groups (Figures 4A,B). In quantification, sub-diploid DNA augment was observed in the cisplatin mono treatment group, and an increase in the G2/M phase to groups 1:1 and 1:3 of combined treatments was also detected. In addition, an increase in the S phase of cisplatin and 1:3 combined treatment and a decrease in the G0/G1 phase for the mono treatment with cisplatin and combined treatments (P:C) were found in all the proportions investigated. The increase in the G2/M phase was interpreted as indicative of the fact that a considerable population was arrested in this cell cycle phase.

### 3.4 Acridine Orange Assay Indicated the Formation of Acid Vacuole Organelles

**FIGURE 5:**
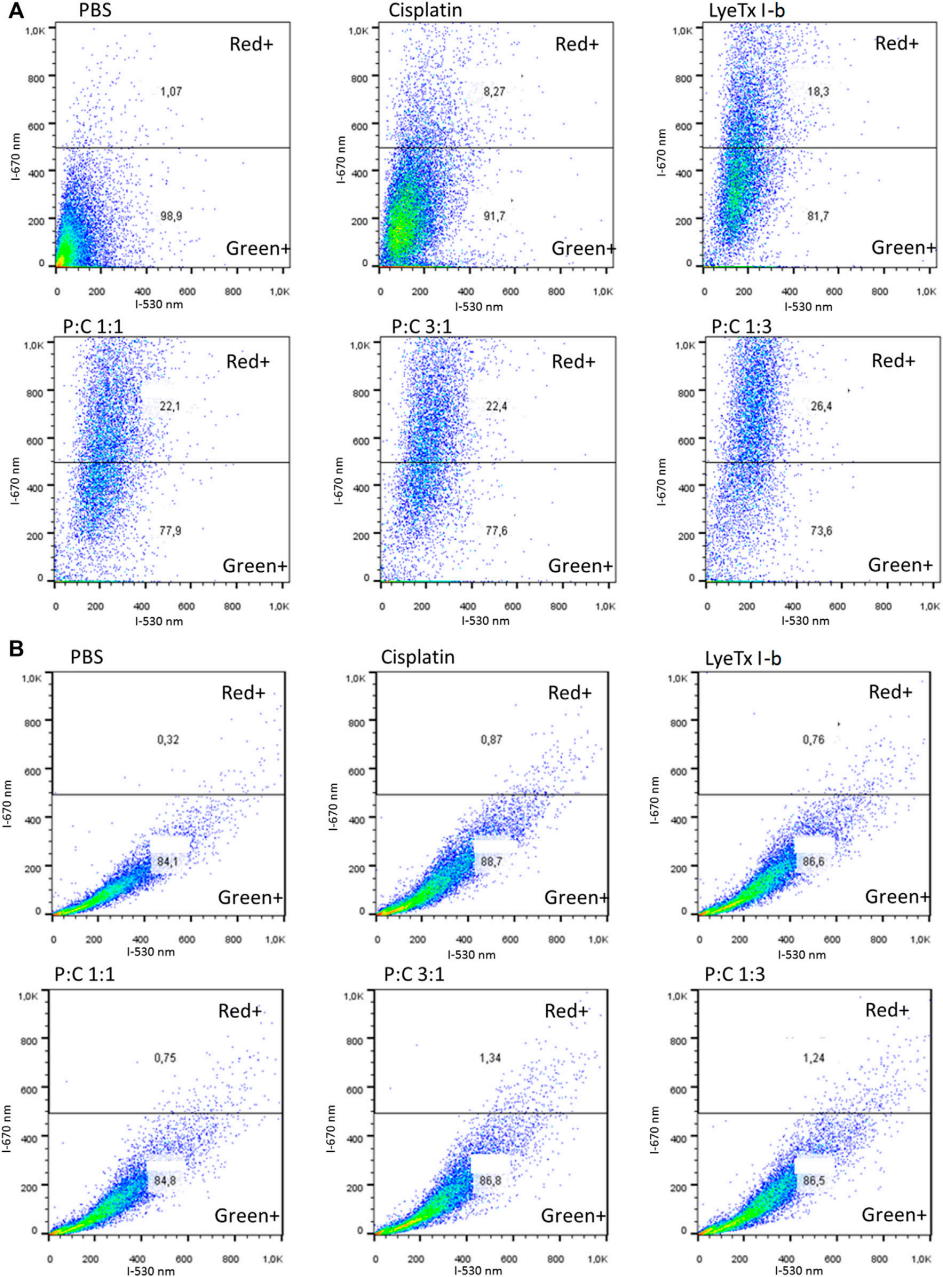
Flow cytometry representative profiles of acid vacuolar organelles after 48 h of each treatment for their respective IC_50_ values against MDA-MB-231 cells analyzed by acridine orange fluorescent stain. **(A)** Results obtained show the population shift of all treatments, especially to LyeTx I-b and the combined treatments. **(B)** Representative results show the population shift ablation with the previous pretreatment with bafilomycin A1, an ATPase proton pump inhibitor.

**FIGURE 6:**
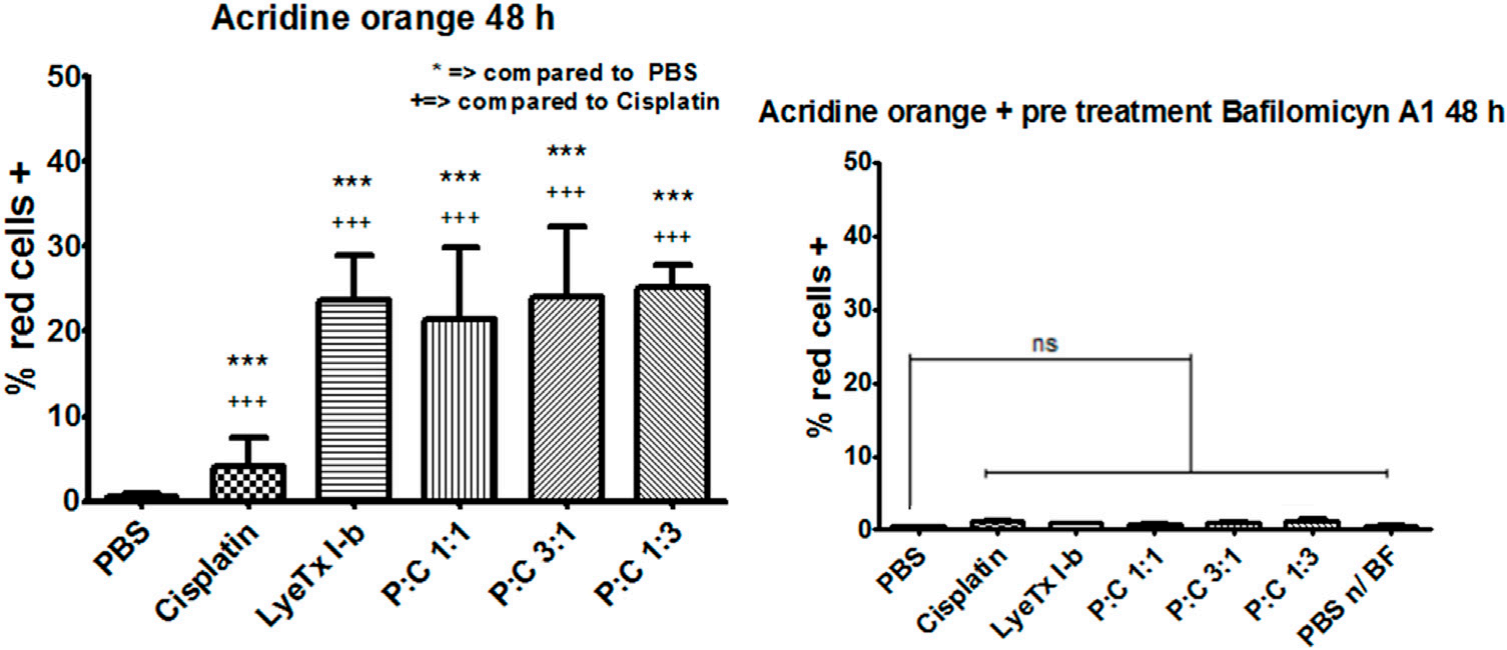
Acridine orange fluorescent stain quantification (*n* = 4) of red cells (MDA-MB-231) marked by this dye after 48 h of each treatment for their respective IC_50_values. **(A)** Quantification results for each treatment with the statistics as specified in the figure. **(B)** Quantification results for each treatment with the previous pretreatment with bafilomycin A1, an ATPase proton pump inhibitor. ***,+++ *p* < 0.001 and “ns”: not significant.

The evaluation of autophagy by acid vacuole organelles was performed using metachromatic acridine orange dye. This dye displays green fluorescence at neutral pH and red at acidic pH. The assay demonstrated that, in all treatments, there was an increase of red organelles (Figure 5A), which was higher in the LyeTx I-b and combined treatments than in the cisplatin group, as stated by the statistical test (Figure 6A).

Pre-treatments with bafilomycin A1 showed that an increase in all red was ablated, decreasing to the control levels as observed in Figure 5B and quantifications as seen in Figure 6B, suggesting a vacuolar pH influence on the redshift.

### 3.5 AKT, ERK, P53, and P21 Immunoblotting Patterns Post-Treatment

**FIGURE 7:**
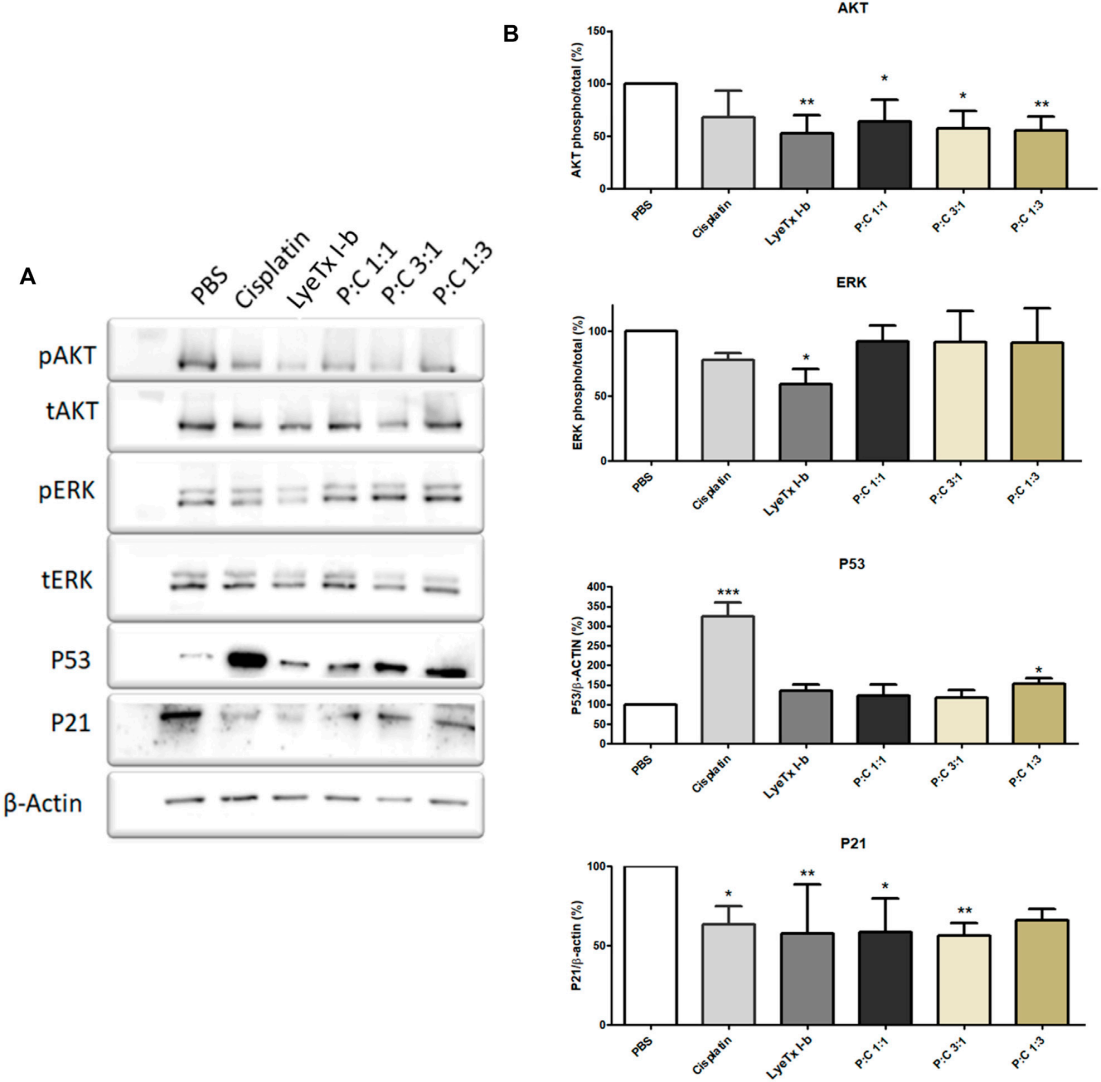
Effects of each treatment (for their respective IC_50_ values) against MDA-MB-231 cells analyzed after 24 h by Western blot. For AKT and ERK, the results show the phosphorylation pattern of these proteins to each treatment. For P53 and P21, the results show the expression pattern of these proteins. **(A)** A representative Western blot shows the pattern found. **(B)** Quantification of the proteins investigated (n = 4). * *p* < 0.05; ** *p* < 0.01; *** *p* < 0.001.

Immunoblots showed a decrease of AKT phosphorylation in the LyeTx I-b and combined treatments groups, as well as a reduction of P21 expression in all treatments. On the other hand, ERK phosphorylation decreased after LyeTx I-b treatment, and P53 was increased in the groups treated with cisplatin and a combination of P:C (1:3) (Figures 7A,B).

## 4 Discussion

Cisplatin is a well-based therapy in clinics (Fleming et al., 2004; Silver et al., 2010; Valle et al., 2014; Wahba and El-Hadaad, 2015) despite its strong side effects, such as nephrotoxicity, vomiting, ototoxicity, myelotoxicity, and neuropathy (Kottschade et al., 2016; Starobova and Vetter, 2017; Manohar and Leung, 2018). For these reasons, combined therapy, an alternative that has been previously studied in basic research and clinical trials, could be helpful to prevent or decrease these clinical side effects (Fleming et al., 2004; Su et al., 2014; Qiao et al., 2018). The peptide LyeTx I-b has been shown to have good anticancer activity in glioblastoma cells (Abdel-Salam et al., 2019) and in a 4T1 mouse mammary carcinoma model (Abdel-Salam et al., 2021). It is noteworthy that the tumor, metastases, and nodules were decreased in LyeTxI-b-treated animals, and an area of necrosis was visible at the site of the treated tumors.

In the present work, we confirmed the activity of LyeTxI-b against breast cancer cells (MDA-MB-231) and evaluated the possibility of this peptide improving the activity of cisplatin. The IC_50_ values found for LyeTxI-b against MDA-MB-231 and HEK-293 cells were 2.47 and 7.88 µM, respectively. It shows apparent lower toxicity of the peptide in the control cells (HEK-293) compared to cancer cells, while cisplatin did not show selectivity in the breast cancer cells used in this study. However, it should be more explored and assayed in other noncancer cells. A combined treatment using this peptide and cisplatin against MDA-MB-231cells resulted in better selectivity index (SI) values for the combinations 1:1 (SI of 0.67) and 3:1 (0.85) (peptide:cisplatin) compared to cisplatin, the chemotherapy established in clinics. This finding is important due to the need to use a well-established drug with new testing drugs, such as the peptide LyeTxI-b. The SI values found for the combinations 1:1 and 3:1 were higher, 16 and 20 times, respectively, than cisplatin (SI = 0.041). Moreover, the proportion 1:1 presented a synergistic effect, as observed in the isobolographic analysis, indicating that this combination, unlike cisplatin alone, seems to be a promising option for advanced trials *in vivo*.

Since one of the worst side effects of cisplatin is nephrotoxicity (Starobova and Vetter, 2017), the SI values of the combinations 1:1 and 1:3 suggest that the combination presented in this study could be less nephrotoxic than that caused by the cisplatin monotherapy treatment. This decrease in toxicity is also based on the non-tumoral cell model (HEK-293), which originated from a fetal kidney, used as our toxicity control. Nephrotoxicity occurs in 20–30% of patients treated with cisplatin (Miller et al., 2010), and this phenomenon is increased by aging (Liu et al., 2018), being a significant issue in most patients treated for breast cancer, a common pathology in aged women. The isobolographic analyses showed that the best combination seems to be 1:1, with synergistic effect among the drugs, good SI, and using about 48 times less cisplatin than that used in monotherapy. Furthermore, LyeTx I-b did not present histopathological alterations in organs such as the spleen, heart, brain, lungs, and kidneys in the studies conducted by Abdel-Salam et al. (2021). This indicates that an *in vivo* trial for the combination of LyeTx I-b with cisplatin would not present significant damages, suggesting fewer side effects when using this combination.

Our data indicate that the combination of LyeTx I-b with cisplatin in the proportion 1:1 against MDA-MB-231 cells provoked a synergistic effect. In contrast, the other two proportions (i.e., 3:1 and 1:3) presented an additive effect. This finding suggests that the cooperation between the drugs to kill MDA-MB-231 cells depends on the proportion of the constituents in the mixture, in which the ratio of 1:1 is the more cooperative among our tested conditions. This is not an uncommon finding for drugs used in combination and have a differential profile of interaction according to the proportion of the constituents (Gessner and Cabana, 1970). Nevertheless, the mechanism behind such differential interaction is elusive and requires further investigation. The synergistic effects of this combined treatment could be explained considering that each compound acts by a different mechanism of action, in which LyeTx I-b acts primarily by a membranolytic mechanism (Santos et al., 2010; Abdel-Salam et al., 2019). In addition, we have recently shown an immunomodulatory effect of this peptide, which significantly decreased the expression of IL-1β in tumor and lung tissues and increased the levels of IL-10 anti-inflammatory cytokines in the primary tumor of 4T1 mouse mammary carcinoma (Abdel Salam et al., 2021) as observed in other AMPs. It is known that IL-10 protects from carcinogenic and improves tumor immune surveillance, besides inhibiting tumor migration and progression, particularly in the early stages of breast cancer (Mumm et al., 2011; Holen et al., 2016). On the other hand, cisplatin damages DNA (Dasari and Tchounwou, 2014). Therefore, the combination of both compounds targeting different sites could increase the efficacy of the treatment of mammary cancer.

The evaluation of a possible mechanism of death for the combination of LyeTx1-b with cisplatin suggested autophagic cell death (ACD). This mechanism occurs by autophagosome vesicles, which engulf the cell organelles leading to an autodigestion process, in some cases, this process can also digest antiapoptotic proteins facilitating cell death cascade, such as apoptosis (Kroemer et al., 2009; Azzopardi et al., 2017; Denton and Kumar, 2019).

The cell cycle revealed a G2/M arrest to the 1:1 and 1:3 combinations of LyeTx I-b with cisplatin. An increase in the G2/M phase is evidence of G2/M arrest, a well-known indicative of ACD (Dotiwala et al., 2013; Azzopardi et al., 2017; Mathiassen et al., 2017). This ACD hypothesis is corroborated by assays with acridine orange that suffers metachromasia in lower pH, emitting fluorescence in the 670 nm range (red). As the autophagosome activity is dependent on the decrease in pH inside the vesicle, it is expected a shift from green to red in cells compromised with autophagic events (Han and Burgess, 2010; Murugan and Amaravadi, 2016). Therefore, as observed in our experiments, all combined treatments and the mono treatment with LyeTx I-b exhibited a significant cell population shift toward the red positive marked area in the analysis. For the combinations of LyeTx I-b with cisplatin (1:1, 3:1, and 1:3), the cell population move toward red positive marked cells was 42.8, 48, and 50.2 times, respectively, compared to the control. This cell population shift is due to the active acid autophagosomes, as previously described (Nagelkerke et al., 2015; Klionsky et al., 2016; Honorato et al., 2020). Additionally, we tested the cells pretreated with bafilomycin A1, a well-known autophagic inhibitor. We observed a reversion in the effects to the control levels, indicating that the red fluorescence is autophagosome-dependent (Shacka et al., 2006; Klionsky et al., 2016; Thomé et al., 2016).

Using the protein markers AKT1, ERK, and P21, we found that the profiles of AKT1 and P21 were modified. The AKT1 phosphorylation reduction is indicative of a catabolic state, and it is related to the autophagic activity (Cao et al., 2015; Shao et al., 2016; Palmieri et al., 2017). Furthermore, in some conditions, when AKT is active, it regulates autophagy suppression (Settembre et al., 2011; Palmieri et al., 2017). The decrease of P21, whose activity is related to separate cyclin CDK complexes (Abbas and Dutta, 2009), could explain the arrest in the G2/M cell cycle. Due to this activity, the decrease in P21 expression is probably related to allowing cells to pass through G1 and S phases and be arrested in G2/M by another P21-independent mechanism. As ERK phosphorylation remains at the control levels, except for a modest LyeTx I-b-induced decrease, this protein appears not to be involved in response to the combined treatment. The importance of this finding is because ERK activity increase is involved in autophagy related to survival and not to autophagic cell death events (Nagelkerke et al., 2015).

In summary, we found that the combination of LyeTx I-b with cisplatin has a better SI value when compared to cisplatin alone. The combination in the proportion 1:1 is synergistic, and the mechanistic studies suggest autophagic cell death as the primary cell death mechanism involved. On the other hand, Abdel-Salam and collaborators found in neuroblastoma cell lines a necroptotic cell death mechanism when these cells were treated with LyeTx I-b peptide (Abdel-Salam et al., 2019), suggesting that the peptide can stimulate different cell death mechanisms, depending on the cell line. Furthermore, in the *in vitro* 4T1 mouse mammary carcinoma model, the effect of this peptide was associated with the induction of apoptosis and inhibition of cell proliferation (Abdel Salam et al., 2021). We suppose that the combination of the peptide LyeTx-I-b with other anticancer drugs could give good results to improve the treatment of other cancer types. *In vivo* experiments will be conducted to validate the efficacy of the combined treatment (peptide:cisplatin) in a model of triple-negative cancer.

## Data Availability

The raw data supporting the conclusions of this article will be made available by the authors, without undue reservation.
